# The effect of continuous interscalene nerve blockade on hospital length of stay following shoulder surgery

**DOI:** 10.1186/1753-6561-6-S4-O31

**Published:** 2012-07-09

**Authors:** C Udovicich, M Richardson, J Cormack

**Affiliations:** 1 The University of Melbourne, Australia; 2 The Epworth Hospital, Australia; 3 The Royal Melbourne Hospital, Australia; 4 The St. Vincent’s Hospital, Australia

## Introduction

Continuous interscalene nerve blockade (CISNB) has been shown to reduce postoperative pain and morphine usage after a wide range of shoulder operations (1&2). Recent trends to minimally invasive shoulder surgery are expected to be associated with shorter postoperative stays. This study compared the length of stay using CISNB as the primary postoperative analgesia with other forms of analgesia and measured the time from end of surgery to discharge.

## Methods

Following ethics committee and institutional approval from the participating hospital (The Epworth Hospital, Richmond), the investigator examined the medical records of all patients having minimally invasive shoulder surgery undertaken by the one surgeon. Included were shoulder acromioplasty, shoulder capsulotomy, superior labral anterior posterior (SLAP) repair and rotator cuff repair (mini-open). Exclusions were patients under 16 years, ASA status greater than 3, bilateral surgery and emergency cases.

## Results

There was no difference between groups with median hospital length of stay from the end of surgery to discharge being 24.4 hours (quartiles 20.7-42.1) for the CISNB (A) group compared with 24.0 hours (18.8-42) for the “other analgesia “(B) group which included parenteral morphine alone or in combination with single shot interscalene nerve block or intra-articular local anaesthetic. Secondary observed outcomes included opioid use on the ward in morphine equivalents with CISNB patients requiring a median dose of 2mg (quartiles 0-9) compared with “other analgesia” needing 6mg (1-16) p=<0.01.

## Conclusions

For minimally invasive shoulder surgery the presence of CISNB is not associated with shortened hospital stay despite requiring less morphine equivalents. Further analysis of patients with stays over 48 hours showed rotator cuff repairs and age over 65 may be associated with longer stay however this study was underpowered to prove this association.

